# Anti-Cancer Activity of As_4_O_6_ and its Efficacy in a Series of Patient-Derived Xenografts for Human Cervical Cancer

**DOI:** 10.3390/pharmaceutics12100987

**Published:** 2020-10-19

**Authors:** Joseph J. Noh, Myeong-Seon Kim, Young-Jae Cho, Soo-Young Jeong, Yoo-Young Lee, Ji-Yoon Ryu, Jung-Joo Choi, Illju Bae, Zhaoyan Wu, Byoung-Gie Kim, Jae Ryoung Hwang, Jeong-Won Lee

**Affiliations:** 1 Division of Gynecologic Oncology, Department of Obstetrics and Gynecology, Samsung Medical Center, Sungkyunkwan University School of Medicine, Seoul 06351, Korea; josephnoh.medicine@gmail.com (J.J.N.); sy1130.jeong@samsung.com (S.-Y.J.); yooyoung.lee@samsung.com (Y.-Y.L.); 2 Department of Obstetrics and Gynecology, St. Vincent’s Hospital, Catholic University of Korea, Seoul 16247, Korea; mseon.kim@outlook.com; 3 Research Institute for Future Medicine, Samsung Medical Center, Sungkyunkwan University School of Medicine, Seoul 06351, Korea; yj35.cho@sbri.co.kr (Y.-J.C.); jiyoon.ryu@sbri.co.kr (J.-Y.R.); jungjoo.choi@samsung.com (J.-J.C.); 4 Chemas Co., Ltd., Seoul 06163, Korea; cjsbij@gmail.com (I.B.); pineapple97531@gmail.com (Z.W.)

**Keywords:** cervical cancer, tetraarsenic hexoxide, patient-derived xenograft, autophagy, cisplatin

## Abstract

Purpose: To investigate the anti-cancer effects of tetraarsenic hexoxide (TAO, As_4_O_6_) in cervical cancer cell lines and in a series of patient-derived xenograft (PDX) mouse models. Methods: Human cervical cancer cell lines, including HeLa, SiHa and CaSki, and human umbilical vein endothelial cells (HUVECs), were used to evaluate the anti-cancer activity of TAO. Cellular proliferation, apoptosis, and enzyme-linked immunosorbent assay (ELISA) for matrix metallopeptidase 2 (MMP-2) and 9 (MMP-9) were assessed. The tumor weights of the PDXs that were given TAO were measured. The PDXs included primary squamous cell carcinoma, primary adenocarcinoma, recurrent squamous cell carcinoma, and recurrent adenocarcinoma. Results: TAO significantly decreased cellular proliferation and increased apoptosis in cervical cancer cell lines and HUVEC. The functional studies on the cytotoxicity of TAO revealed that it inhibited the activation of Akt and vascular endothelial growth factor receptor 2 (VEGFR2). It also decreased the concentrations of MMP-2 in both cervical cancer cell lines and HUVECs. Active caspase-3 and p62 were both increased by the treatment of TAO, indicating increased rates of apoptosis and decreased rates of autophagy, respectively. In vivo studies with PDXs revealed that TAO significantly decreased tumor weight for both primary squamous cell carcinoma and adenocarcinoma of the cervix. However, this anti-cancer effect was not seen in PDXs with recurrent cancers. Nevertheless, the combination of TAO with cisplatin significantly decreased tumor weight in PDX models for both primary and recurrent cancers. Conclusions: TAO exerted inhibitory effects on angiogenesis, cellular migration, and autophagy, and it showed stimulatory effects on apoptosis. Overall, it demonstrated anti-cancer effects in animal models for human cervical cancer.

## 1. Introduction

Cervical cancer is one of the most common cancers among women worldwide, with almost half a million new cases occurring in each year. In 2015, 526,000 women were diagnosed with cervical cancer and the estimated number of deaths caused by the disease was 239,000 [1]. Cervical cancer patients are prone to developing pelvic recurrence or distant metastasis. A 10–20% recurrence rate has been reported following primary surgery or radiotherapy in women with stage IB–IIA cervical cancer with no evidence of lymph node involvement, while up to 70% of patients with nodal metastases were reported to relapse [2,3]. Because of the unfavorable prognosis of the disease and its high recurrence rate, cervical cancer continues to be a major public health problem, despite widespread screening methods [4]. Various treatment modalities including chemotherapy and radiotherapy have been developed, but none have demonstrated promising results thus far. In order to develop a new approach to improve the prognosis of cervical cancer, researchers have started to investigate various non-chemotherapeutic agents, such as arsenic trioxide (As_2_O_3_) and tetraarsenic hexoxide (As_4_O_6_, TAO).

Arsenic is a naturally occurring substance that has been used as a medicinal agent for more than 2400 years to treat a variety of medical conditions ranging from infectious disease to cancer [5]. It is stable in dry air, but the surface oxidizes slowly in moist air to give a bronze tarnish and, finally, a black covering to the element. When heated in the air, it ignites to form arsenic trioxide and tetraarsenic hexoxide. In traditional Chinese medicine, arsenic trioxide is recorded in the Compendium of Materia Medica as having therapeutic benefits. Because of the toxic side effects and the introduction of modern radiotherapy and chemotherapy, Western medicine has abandoned the use of arsenic as a treatment for cancer. However, its therapeutic effect on leukemia has initiated a re-awakening of interest in arsenic compounds. Studies have demonstrated that TAO induces apoptosis in hematopoietic and non-hematopoietic tumor cells, eventually gaining approval from the U.S. Food and Drug Administration (FDA) to treat acute promyelocytic leukemia (APL) [6]. The molecular formula of the drug substance in the solid state is As_2_O_3_ (molecular weight of 197.84 g mol^−1^). Because arsenic trioxide is poorly soluble in pure water, inactive ingredients, such as sodium hydroxide, are added to increase its solubility. Under normal conditions (room temperature and atmospheric pressure), solid arsenic trioxide is present in the form of As_4_O_6_ (dimeric As_2_O_3_) and only dissociates into monomeric As_2_O_3_ above 800 °C. Upon dissolution of arsenic trioxide in aqueous media, both As_2_O_3_ and As_4_O_6_ are converted into the same arsenic species. It is hypothesized that its anti-cancer effects are mediated by the induction of cellular differentiation, tumor cell apoptosis, degradation of specific transcripts, inhibition of tumor cell growth, modulation of redox balance, and abrogation of vascular networks that cause blood flow to shut down, subsequently causing cell necrosis [7,8,9,10]. In an effort to add further evidence to the body of literature suggesting the anti-cancer effects of TAO, the present study was designed to demonstrate the effectiveness of TAO in a series of patient-derived xenografts (PDXs) for cervical cancer, including primary and recurrent patients.

## 2. Materials and Methods

### 2.1. Cell Lines and Tetraarsenic Hexoxide

Three different cervical cancer cell lines, SiHa (*Homo sapiens* uterine cervix, squamous cell carcinoma: HTB-35), HeLa (*Homo sapiens* uterine cervix, adenocarcinoma: CCL-2), CaSki (*Homo sapiens* uterine cervix, derived from metastatic site of small intestine, epidermoid carcinoma: CRL-1550), and human umbilical vein endothelial cells (HUVECs, CRL-1730) were obtained (American Type Culture Collection, Manassas, VA, USA). Cells were maintained in Dulbecco’s modified eagle medium (DMEM), minimal essential medium (MEM), and Roswell Park Memorial Institute (RPMI) medium containing 10% fetal bovine serum (FBS) with 100 units/mL penicillin and 100 µg/mL streptomycin (Invitrogen, Carlsbad, CA, USA) for HeLa, SiHa, and CaSki, respectively. They were grown at 37 °C in a 5% CO_2_ incubator. HUVECs were grown in an endothelial cell growth medium 2 (EGM-2) bullet kit (Lonza, Basel, Switzerland). TAO was obtained from CHEMAS (Seoul, South Korea). A 1% concentration of TAO solution in distilled water was made by heating for 4 h at 90–100 °C and was then filtered through a 0.2 µm filter.

### 2.2. MTT (3-(4,5-Dimethylthiazol-2-yl)-2,5-diphenyltetrazolium bromide) Assay

For assaying cell viability, cells were plated with 3000–4000 cells/well onto a 96-well plate in triplicate and then treated with the indicated amount of TAO for 72 h at 37 °C in a 5% CO_2_ incubator. After the drug treatment, cells were incubated with 5 mg/mL MTT (3-(4,5-Dimethylthiazol-2-yl)-2,5-diphenyltetrazolium bromide, M2128, Sigma-Aldrich, St. Louis, MO, USA) solution in 1X phosphate-buffered saline (PBS) for 4 h in a 37 °C incubator. The MTT crystal was dissolved in dimethyl sulfoxide (DMSO, D1370, Duchefa Biochemie, Haarlem, The Netherlands), and cell viability was measured at 540 nm by spectrometry.

### 2.3. Western Blot Analysis

Cells were plated with 200,000 cells/well on a 6-well plate. The cells were treated with TAO at concentrations indicated in the text for 48 h. Cellular protein was lysed by incubating for 20 min on ice in radioimmunoprecipitation assay (RIPA) buffer containing 1X protease inhibitor mix (P-8340, Sigma-Aldrich, St. Louis, MO, USA) and 1 mmol/L of phenylmethylsulfonyl fluoride (PMSF, P-7626, Sigma-Aldrich, St. Louis, MO, USA). Protein concentrations were determined using the Bio-Rad protein assay (Bio-Rad Laboratories, Hercules, CA, USA), and proteins were separated by sodium dodecyl sulfate-polyacrylamide gel electrophoresis (SDS-PAGE), then were electrotransferred to a polyvinylidene difluoride (PVDF) membrane. After blocking membranes with 5% non-fat dry milk in PBS, membranes were incubated with primary antibodies overnight at 4 °C. After several washes, blots were incubated with secondary antibodies (GeneTex, Irvine, CA, USA) for 1 h. After an additional wash, light development was initiated by adding enhanced chemiluminescence (ECL) reagents (Amersham PLC, Buckinghamshire, United Kingdom). Primary antibodies for studying phosphorylated Akt (Ser473, #9271), Akt (#9272), and VEGFR2 (#2479) were obtained from Cell Signal Technology (Danvers, MA, USA), and β-actin (sc-47778) was obtained from Santa Cruz Biotechnology (Dallas, TX, USA). Phosphorylated-vascular endothelial growth factor receptor 2 (VEGFR2, ab5473) and p62 (ab56416) were obtained from Abcam (Cambridge, UK), and anti-LC3 antibody (NB100-2220) was purchased from Novus Biologicals (Centennial, CO, USA).

### 2.4. ELISA (Enzyme-Linked Immunosorbent Assay) for MMP-2 (Matrix Metallopeptidase 2) and 9

For the enzyme-linked immunosorbent assay (ELISA), media was collected from the cells in culture and transferred to a 96-well plate for matrix metallopeptidase 2 (MMP-2, MMP200) or MMP-9 (DMP900) specific ELISAs using a Quantikine ELISA Kit (R&D Systems, Minneapolis, MN, USA) according to the manufacturer’s instructions. A standard curve using each recombinant protein provided in the kit was run with each assay, and the concentration of each protein was determined by the standard curve.

### 2.5. Caspase-3 Assay

Cell death was assessed using an active caspase-3 assay kit (KHO1091, Invitrogen, Carlsbad, CA, USA). Cells (2 × 10^5^/well) were plated in a 6-well plate and treated with TAO as indicated for 48 h. After TAO treatment, cells were lysed in RIPA buffer, and 50 ug of total protein was used for the caspase-3 assay according to the manufacturer’s instructions.

### 2.6. Treatment of VEGF to Cervical Cancer Cell Lines and HUVEC

Cells (2 × 10^5^/well) were plated in a 6-well plate and treated with TAO in the growth media as indicated for 48 h. Human vascular endothelial growth factor (VEGF165, H9166) was purchased from Peprotech (Rocky Hill, NJ, USA) and was dissolved in 1X PBS containing 0.1% bovine serum albumin (BSA, A-3294, Sigma-Aldrich, St. Louis, MO, USA) to make a 100 μg/mL solution. VEGF was introduced into the growth media containing TAO overnight before lysis of the cells.

### 2.7. Animal Study Using Cell Lines and PDX (Patient-Derived Xenograft) Models for Cervical Cancer

To establish the SiHa cell line xenograft tumor, female BALB/c nude mice were purchased from Orient Bio (Seongnam, South Korea). Autoclaved water and food were available to the mice *ad libitum*. The same SiHa cell line that was used for in vitro experiments was employed. It was cultured in MEM containing 10% FBS. A total of 2 × 10^6^ cells were inoculated subcutaneously in 200 µL of Hanks’ Balanced Salt Solution (HBSS, Biocompare, San Francisco, CA, USA) into the flank of the animals bilaterally. Tumor growth was measured twice a week. The volume of tumors was calculated using a standard formula (length × width^2^ × 0.52), and growth curves were drawn.

To establish PDX models of cervical cancer, surgically removed patient tumor specimens were reduced to small pieces (less than 2–3 mm), implanted into the subrenal capsules of the left kidneys of BALB/c nude mice, and propagated by serial transplantation [11,12]. The clinical information of the patients is provided in Supplementary Table S1 and Supplementary Figures S1–S5. The mice used in these experiments were 6 to 8-weeks old. TAO (8 mg/kg) or PBS was intraperitoneally injected into the model mice once a week for the subsequent 3–4 weeks. For combination therapy with cisplatin, the model mice were either given intraperitoneal cisplatin (4 mg/kg) once a week alone or cisplatin (same dosage) in addition to TAO injections. The mice were then sacrificed and tumors were imaged and weighed. The mice (*n* = 10 per group) were monitored daily for tumor development and sacrificed when any appeared moribund. We recorded the body weight, tumor weight, and number of tumor nodules. Tumors were fixed in formalin and embedded in paraffin or snap-frozen in optimal cutting temperature (OCT) compound (Sakura Finetek Japan, Tokyo, Japan) in liquid nitrogen. This study was reviewed and approved by the Institutional Animal Care and Use Committee (IACUC) of the Samsung Biomedical Research Institute (protocol number H-A9-003). The IACUC is accredited by the Association for the Assessment and Accreditation of Laboratory Animal Care International (AAALAC International) and abides by the guidelines of the Institute of Laboratory Animal Resources (ILAR). Study of the PDX model for cervical cancers was approved by the Samsung Medical Center Institutional Review Board (IRB file number 2010-04-004) and experiments were performed in accordance with the approved guidelines and regulations.

### 2.8. Statistical Analysis

The Mann–Whitney U test was used to compare differences between the groups in both in vitro and in vivo assays. All statistical tests were two-sided, and *P* values less than 0.05 were considered to be statistically significant. SPSS software (Version 17.0; SPSS, Chicago, IL, USA) was used for all statistical analyses.

## 3. Results

### 3.1. Effects of TAO on Cell Viability, Apoptosis, and Cell Line Xenograft

**Figure 1:**
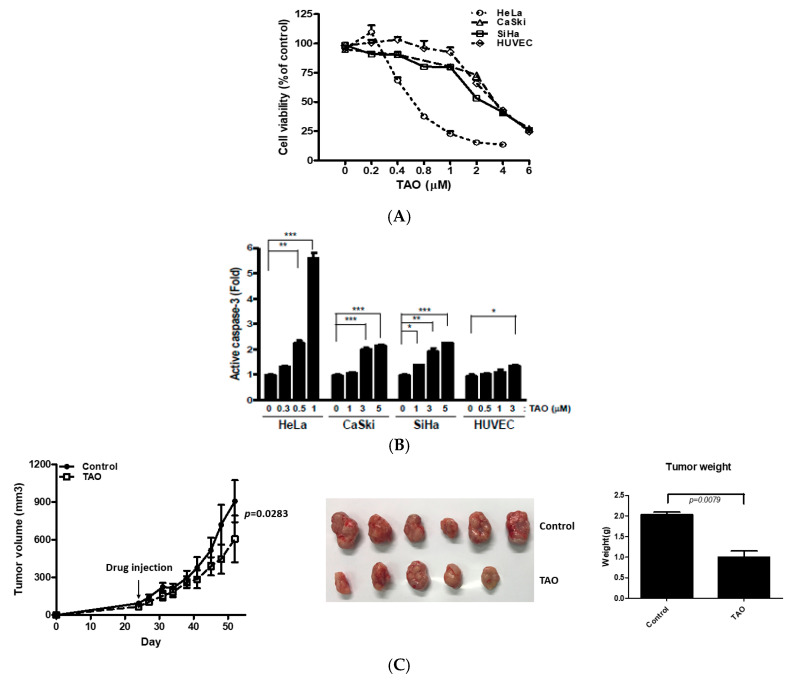
(**A**) Cellular metabolic activities in cervical cancer cell lines and human umbilical vein endothelial cells (HUVECs) measured by MTT assay. Decreasing cell viability with increasing concentration of tetraarsenic hexoxide (TAO) is shown; (**B**) Increasing levels of active caspase-3 are shown in cervical cancer cell lines and HUVECs as the concentration of TAO increases; (**C**) Significant difference is observed in tumor volumes between the mice bearing SiHa cells treated with TAO vs. 0.9% sodium chloride solution. The days represent post-implantation of tumor cells. TAO or 0.9% sodium chloride solution was injected once the tumor grew to a certain volume, which was about 85.5 mm^3^ in the present experiments. The mean weight of the treatment group vs. control group shows a statistically significant difference. * *p*-value < 0.05, ** *p*-value < 0.01, *** *p*-value < 0.001.

In order to see the effects of TAO on cell viability, an MTT assay was performed in cervical cancer cell lines, including SiHa, HeLa, CaSki, and HUVECs. In all three cervical cancer cell lines and HUVECs, cell viability decreased as the concentration of TAO increased (Figure 1A). The IC_50_ of TAO on SiHa, CaSki, and HUVEC at 72 h was 3 µM, and the IC_50_ of HeLa was 0.6 µM (Table 1). The levels of active caspase-3 were also measured with Western blot. It was shown that active caspase-3 increased as the concentration of TAO increased, suggesting an increase of cellular apoptosis in all three cancer cell lines and HUVECs (Figure 1B). Mice bearing SiHa cell tumors were treated with TAO, which was injected intraperitoneally at 8 mg/kg body weight per injection, once a week. Calculated tumor volume was significantly smaller in animal models that were injected with TAO in comparison to those injected 0.9% sodium chloride control solution (Figure 1C).

### 3.2. Effects of TAO on MMP-2 and MMP-9

**Figure 2:**
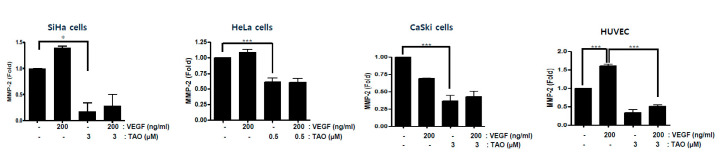
The concentrations of MMP-2 by enzyme-linked immunosorbent assay (ELISA). The treatment of TAO significantly decreased the concentrations of MMP-2 in all cervical cancer cell lines and HUVECs. The co-administration of vascular endothelial growth factor (VEGF) and TAO did not change the inhibitory effects of TAO on MMP-2, whereas the treatment of VEGF only mildly increased the concentrations of MMP-2 in SiHa and HeLa cell lines and HUVECs. * *p*-value < 0.05, *** *p*-value < 0.001.

MMP-2 and MMP-9 were measured by ELISA to examine the inhibitory effects of TAO on extracellular matrix degradation and presumably cancer metastasis (Figure 2). The reduction of MMP-2 was observed by the treatment of TAO in SiHa cell lines. Compared to the expression levels of MMP-2 in SiHa cells that were neither treated by TAO nor VEGF, TAO reduced MMP-2 by about 80%. When SiHa cell lines were treated by both TAO and VEGF, the levels of MMP-2 also decreased significantly. Similar patterns were observed in HeLa and CaSki cell lines. The reduction of MMP-2 was observed by the treatment of TAO. Compared to the expression levels of MMP-2 in each cancer cell line that was not treated by either TAO or VEGF, TAO reduced MMP-2 by about 40% in HeLa and 60% in CaSki cell lines. When CaSki cell lines were treated with VEGF alone, MMP-2 was also reduced, but the reduction was not statistically significant. However, when they were treated with VEGF and TAO together, they showed approximately 25% additional reduction of MMP-2. When HeLa cell lines were treated with VEGF alone, MMP-2 increased by about 5%. However, when they were treated with VEGF and TAO together, they showed 50% reduction in MMP-2. When HUVECs were treated with VEGF, MMP-2 increased by more than 50%. However, the levels of MMP-2 significantly decreased when it was treated with TAO and VEGF together. When HUVECs were treated with TAO without VEGF, MMP-2 was also reduced by about 60%. The extent of reactivity to VEGF seemed to differ among cancer cell lines. However, it was evident that TAO reduced the expression levels of MMP-2, regardless of the seemingly different reactivity of cell lines to VEGF. We could not measure MMP-9 in cancer cell lines as it seemed that MMP-9 was too low to be detected in these cell lines.

### 3.3. Effects of TAO on the Activation of Akt

**Figure 3:**
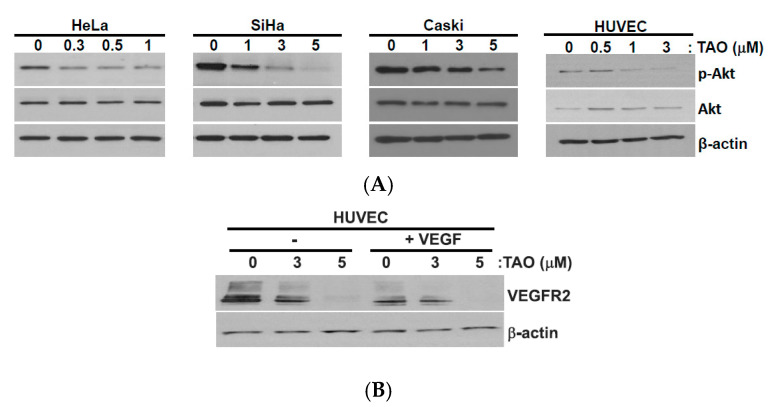
(**A**) Western blot analysis of Akt and phosphorylated-Akt (p-Akt) in the cell lines 48 h after the treatment of TAO with varying concentrations. The treatment of TAO decreases the phosphorylation of Akt. (**B**) Western blot analysis of vascular endothelial growth factor receptor 2 (VEGFR2), demonstrating that the treatment of TAO in HUVECs results in decreased concentration of VEGFR2. The same pattern of observation is seen in HUVEC, both with and without the treatment by vascular endothelial growth factor (VEGF).

Akt (also known as protein kinase B (PKB)) is a serine/threonine protein kinase. Akt plays an important role in intracellular signaling pathways that are involved in glucose metabolism, apoptosis, cell proliferation, DNA transcription, and cell migration. It promotes cancer cell invasion by increasing motility and metalloproteinase production [13]. Western blot analysis of Akt and phosphorylated-Akt (p-Akt) were performed after the treatment of cervical cancer cell lines with TAO for 48 h. The results demonstrated that the levels of p-Akt decreased dose-dependently with the treatment of TAO in all HeLa, SiHa, and CaSki cell lines and HUVECs, suggesting the inhibitory effects of TAO (Figure 3A). The levels of total Akt, however, were not affected by the treatment of TAO and remained constant in all cancer cell lines and HUVECs as the concentration of TAO treatment increased.

### 3.4. Effects of TAO on VEGF-Related Signaling Pathway

HUVECs are known to be sensitive to vascular endothelial growth factor (VEGF). There are three receptor types of the VEGF signaling pathway, and VEGF receptor 2 (VEGFR2) is the major one among them. When the signal is initiated by VEGF, VEGFR2 is auto-phosphorylated, and the corresponding pathway is activated. To investigate the inhibiting effects of TAO on angiogenesis, we studied the response of VEGFR2 in HUVEC to TAO.

HUVECs were treated with TAO for 24 h, followed by Western blot analysis. As the concentration of TAO increased, the expression levels of VEGFR2 decreased. The same results were seen when the concurrent treatment of VEGF was performed (Figure 3B). These findings suggest that TAO suppresses the expression of VEGFR2, thereby exerting its inhibitory effects on the signaling pathway that is related to angiogenesis, regardless of the presence of signaling molecules.

### 3.5. Effects of TAO on Autophagy

**Figure 4:**
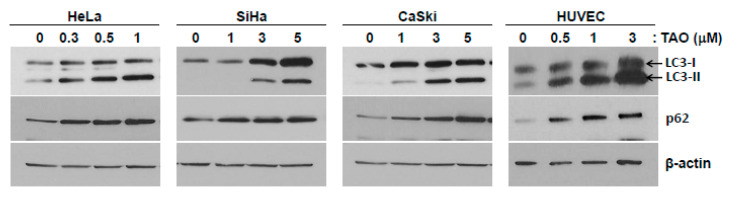
Western blot analysis of autophagy-related proteins in cervical cancer cell lines and HUVECs. The conversion of LC3-II increases as the concentration of TAO increases. Increasing concentrations of p62 are seen as concentrations of TAO increase, suggesting the inhibitory effects of TAO on autophagy.

To determine if TAO was involved in the autophagy pathway, p62, an intermediate of autophagy, and LC3, an autophagy marker protein, were analyzed by Western blot (Figure 4). It was shown that p62 increased with the treatment of TAO in all three cervical cancer cell lines. This implies that autophagy was reduced in the cell lines by TAO. The treatment of TAO also increased the concentrations of p62 in HUVECs. Conversion of LC3-I to LC3-II, a phosphatidylethanolamine-conjugated form of LC3-I, is a marker for autophagy activation. When autophagy is inhibited, LC3-II cannot be degraded by autolysosome and thereby accumulates in the cytoplasm. In cervical cancer cell lines and HUVECs, the accumulation of LC3-II was observed as the concentration of TAO increased, suggesting its inhibitory effects on autophagy.

### 3.6. Effects of TAO in Patient-Derived Xenograft (PDX) Mouse Models

**Figure 5:**
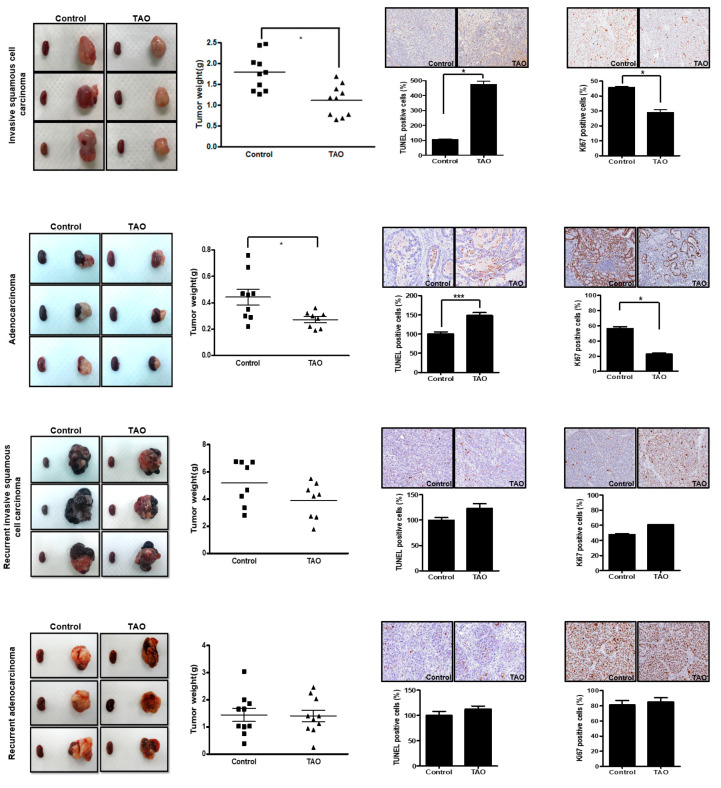
Gross appearance of xenograft from mouse models with primary invasive squamous cell carcinoma, adenocarcinoma, recurrent invasive squamous cell carcinoma, and recurrent adenocarcinoma of the cervix. The tumors of the control group (**left**) vs. the TAO-treated (8 mg/kg) experimental group (**right**) are shown, demonstrating statistically significant differences of tumor weight between the two groups in the primary cancer models. However, this difference is not seen in recurrent cancer models. The TUNEL assay and Ki-67 assay also show the anti-cancer effects of TAO in primary cancer models, while these effects are not seen in recurrent cancer models. * *p*-value < 0.05, *** *p*-value < 0.001.

Patient-derived xenograft (PDX) mouse models of invasive squamous cell carcinoma, adenocarcinoma, recurrent squamous cell carcinoma, and recurrent adenocarcinoma of the uterine cervical cancer were examined. Ten mice were treated with TAO (experimental group), while another 10 mice did not receive the treatment (control group). The mean tumor weight of the control group was measured. In the analysis of primary invasive squamous cell carcinoma PDX, the mean tumor weight of the control group was 2.31 g, while the mean tumor weight of the experimental group was 1.03 g (2.31 ± 1.52 vs. 1.03 ± 0.59, *p* = 0.0232), showing a statistically significant difference between the two groups (Figure 5). The same results were obtained in the PDX mouse models of adenocarcinoma. The mean tumor weight of the control group was 0.44 g, while the mean tumor weight of the treatment group was 0.30 g, with a statistical difference between the two groups (0.44 ± 0.18 vs. 0.30 ± 0.11, *p* = 0.036). The mean tumor weight of the control group in the analysis of recurrent squamous cell carcinoma was 4.43 g, and the mean tumor weight of the experimental group was 3.89 g (4.43 ± 2.17 vs. 3.89 ± 1.33, *p* > 0.05). No statistical difference was observed between the two groups in the recurrent squamous cell carcinoma models. The same experiments were also performed for recurrent adenocarcinoma. The mean tumor weight of the control group was 1.45 g, and the mean tumor weight of the experimental group was 1.41 g (1.45 ± 0.77 vs. 1.41 ± 0.68, *p* > 0.05). Recurrent tumors seemed to have gained resistance to cytotoxic agents and did not respond to the treatment of TAO. The terminal deoxynucleotidyl transferase dUTP nick end labeling (TUNEL) assay was performed to measure DNA fragmentation generated during apoptosis, as described previously [14]. While TUNEL positive cells increased significantly in the tumors of mouse models treated with TAO in primary squamous cell carcinoma and adenocarcinoma, this observation was not seen in the mouse models of recurrent diseases. The Ki-67 protein, a cellular marker for proliferation, was measured in the tumors of the mouse models as well [15]. As seen in Figure 5, the amount of Ki-67 positive cells significantly decreased after the treatment of TAO in primary cancer models, while it did not change in recurrent cancer models.

### 3.7. Effects of Combination of TAO with Cisplatin in PDX Mouse Models

**Figure 6:**
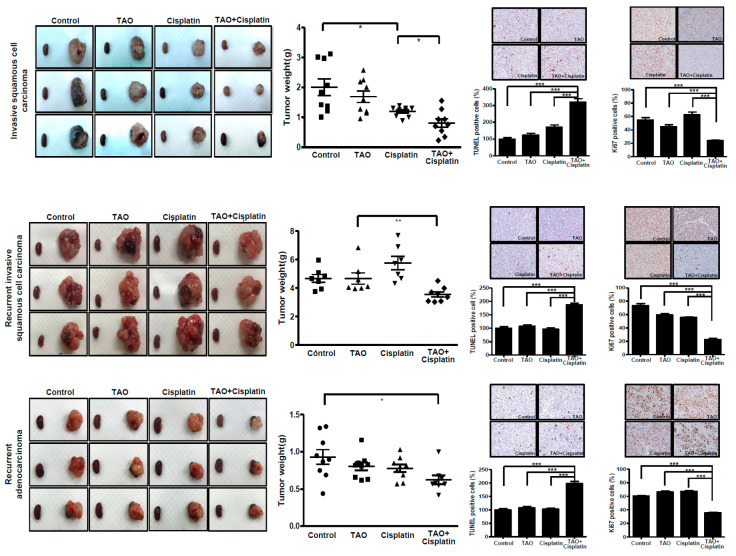
Gross appearance of xenograft from mouse models receiving PBS, TAO (8 mg/kg), cisplatin (4 mg/kg), and TAO plus cisplatin (8 mg/kg of TAO and 4 mg/kg of cisplatin) in order from left to right. Significant reductions of tumor weight in mouse models given TAO plus cisplatin are observed in primary invasive squamous cell carcinoma as well as in recurrent invasive squamous cell carcinoma and adenocarcinoma. The TUNEL assay and Ki-67 assay results also support the synergistic effects of TAO with cisplatin, both in primary and recurrent cancer models. * *p*-value < 0.05, ** *p*-value < 0.01, *** *p*-value < 0.001.

In order to investigate the potential synergistic effects of TAO in addition to conventional cytotoxic agent cisplatin, mouse models were assigned to one of the four groups: control, cisplatin alone, TAO alone, or cisplatin plus TAO. When mouse models with primary invasive squamous cell carcinoma were evaluated, the mean tumor weight of the mouse models that were given cisplatin plus TAO was significantly less than the other three groups (0.94 ± 0.56 vs. 1.84 ± 0.95 vs. 1.30 ± 0.38 vs. 1.61 ± 0.60 g in cisplatin plus TAO, control, cisplatin alone, and TAO alone groups, respectively, *p* = 0.0315). The same patterns of the synergistic anti-cancer effects of TAO with cisplatin were observed in the mouse models generated from recurrent squamous cell carcinoma and recurrent adenocarcinoma (Figure 6). The TUNEL assay and Ki-67 assay were also performed. The synergistic effects of TAO with cisplatin were observed not only in primary cancer models but also in recurrent cancer models.

## 4. Discussion

The present study demonstrated that TAO has anti-cancer effects in patient-derived xenograft mouse models of cervical cancer in vivo, and these results were supported by the experiments with cervical cancer cell lines in vitro. Although the exact mechanisms by which TAO exerts its inhibitory effects on cancer cells is yet to be fully defined, we herein established evidence that it is involved in the regulation of autophagy and angiogenesis of cancer cells to a certain extent. To our knowledge, no previous studies have shown such effects of TAO.

Cervical cancer carries a significant burden on patients, the medical field, and society. Despite widespread screening programs and available vaccinations, it remains one of the most difficult types of cancer to conquer, especially in developing countries. Cervical cancer carries a poor prognosis, and the current standard treatment modalities, especially for advanced stages, do not yield promising clinical results. Although bevacizumab has demonstrated improved survival rates and overall response rates in recurrent cervical cancer in a clinical trial, which has consequently permitted its use as the current standard treatment regimen, further studies to develop efficient and innovative treatment approaches are urgently needed [16].

Preclinical studies have provided some potential therapeutic benefits of TAO on cervical cancer. Studies have shown that it induces cell cycle arrest in the G1 or G2/M phase and suppresses the secretion of MMP-2, which is consistent with the results of the present study. TAO has also been shown to inhibit the phosphorylation of Akt, an upstream signaling protein in MMP-2 and 9 expressions in both cervical cancer cell lines and HUVECs [17]. TAO may also inhibit angiogenesis via downregulation of VEGFR2 expression. In the present study, TAO was shown to inhibit VEGF-induced MMP-2 expression in HUVEC. Because tumor cell metastasis is a complex cascade of events involving multiple steps such as proliferation, adhesion, and migration of cells, an attempt to understand the exact alterations caused by TAO in these intricate relations of different mechanisms is demanding, and there are also other possible mechanisms facilitated within the intricate network of regulatory effects.

Hypoxia and lack of nutrition are a hallmark of cancer tissue, and cancer cells often activate autophagy to evade such conditions. Therefore, autophagy has been considered as one of the survival pathways that growing cancer cells adopt, and its inhibition has been studied for treatment modalities of cancer. The present study demonstrates that the treatment of TAO increases the expression of p62, which normally decreases as the rate of autophagy increases. Although further investigations are warranted to delineate the exact mechanisms of how TAO inhibits autophagy, the evidence shown in the present study provides support for the potential efficacy of TAO as a cancer treatment agent by inducing autophagy. The effects of TAO on the cellular process of autophagy in normal tissue should also be studied because non-specific and global inhibition of autophagy may lead to unwanted clinical consequences.

The interaction of TAO with conventional chemotherapeutic agents and its additive or subtractive effects should also be evaluated. It has been illustrated that arsenic trioxide has a synergistic effect in combination with cisplatin in ovarian cancer cells [18]. Another research group reported the potential synergistic effects of TAO with cisplatin in cervical cancer cells [19,20]. They also reported that the combination of cisplatin with TAO was more effective than the combination of cisplatin with paclitaxel in cervical cancer [20]. The present study suggested the inhibitory effects of TAO on angiogenesis as a possible explanation for the observed results. We also found that the combination of TAO with cisplatin was more effective than cisplatin alone in the PDX mouse model. This effect was seen even in the mouse models with recurrent tumors. Taken together, these results offer further support for the use of TAO in cancer treatment, possibly as a combined regimen with chemotherapeutic agents with previously revealed clinical efficacy.

The lack of significant reduction of cancer tissue in the PDX models of recurrent cervical cancer compared to the substantial decrease in naïve cancer tissue from the TAO treatment is noteworthy. It is thought that the mechanisms by which cells gain resistance to conventional chemotherapeutic agents contribute to the lack of inhibitory effects of TAO seen in recurrent cancer models, and further investigations are necessary.

The strengths of the present study include the utilization of different histology of cervical cancer, such as squamous cell carcinoma, adenocarcinoma, and recurrent cervical cancer. It also employs experiments with cell lines to understand the changes in multiple signaling pathways caused by TAO, as well as xenograft mouse models to validate the eventual consequences of it. One of the limitations of the present study is that it did not evaluate the effects of human papillomavirus (HPV) infection status of tumor cells. Cervical cancer is unique in that most malignant cells are caused by HPV infection. Although TAO has been shown to induce apoptosis of various cancer cell lines, including both HPV-infected and non-infected cells, the mechanisms by which it induces apoptosis may be different [21].

In conclusion, the present study shows that TAO has anti-tumor effects in various types of treatment-naïve cervical cancer cell lines, including squamous cell carcinoma and adenocarcinoma, and these effects are presumably due to its inhibitory effects on autophagy, angiogenesis, and the induction of apoptosis. The potential usefulness of TAO for cervical cancer treatment is thus suggested. Clinical trials of TAO are also suggested to further explore the potential use of TAO in cancer treatment.

## Figures and Tables

**Table 1:** IC_50_ values of TAO on HeLa, CaSki, SiHa cell lines and HUVEC measured by MTT (3-(4,5-dimethyl-thiazol-2-yl)-2,5-Diphenyl-tetrazolium bromide) assay at 72 h after the treatment.

Types of Cell Lines	IC50 (µM)
HeLa	0.6
CaSki	3.0
SiHa	3.0
HUVEC	3.0

## Supplementary Materials

The following are available online at https://www.mdpi.com/1999-4923/12/10/987/s1, Figure S1: T2 sagittal magnetic resonance imaging (MRI) of the cancer lesion that was used to generate the patient-derived xenograft (PDX) I. Figure S2: T2 sagittal MRI of the cancer lesion that was used to generate the PDX II. Figure S3: T2 sagittal MRI of the cancer lesion that was used to generate the PDX III. Figure S4: T2 transverse MRI of the cancer lesion that was used to generate the PDX IV (top). Figure S5: T2 transverse MRI of the cancer lesion that was used to generate the PDX V (top). Table S1: Clinical information of the patients for patient-derived xenograft (PDX).
