# Supplementary material for: Anti-Cancer Activity of As4O6 and its Efficacy in a Series of Patient-Derived Xenografts for Human Cervical Cancer
Source: Pharmaceutics. 2020 Oct 19;12(10):987. doi: 10.3390/pharmaceutics12100987 (PMC7590205; doi:10.3390/pharmaceutics12100987)
Supplement: Supplementary file 1 [file pharmaceutics-12-00987-s001.zip › pharmacuetics 958758 supplementary proof/pharmaceutics-958758 supplementary table.docx]

Anti-Cancer Activity of As_4_O_6_ and its Efficacy in a Series of Patient-Derived Xenografts for Human Cervical Cancer

Joseph J. Noh ^1,†^, Myeong-Seon Kim ^2,†^, Young-Jae Cho ^3,†^, Soo-Young Jeong ^1^,
Yoo-Young Lee ^1^, Ji-Yoon Ryu ^3^, Jung-Joo Choi ^3^, Illju Bae ^4^, Wu Zhaoyan ^4^,
Byoung-Gie Kim ^1,^*, Jae Ryoung Hwang ^3,^* and Jeong-Won Lee ^1,^*

**Table S1.** Clinical information of the patients for patient-derived xenograft (PDX).

| **PDX^a^** | **Histology** | **Age** | **Initial stage^b^** | **Treatment history** |
| --- | --- | --- | --- | --- |
| I | Primary  squamous cell  carcinoma | 46 | IB2 | Radical abdominal hysterectomy and bilateral pelvic lymphadenectomy  Invasive squamous cell carcinoma, keratinizing type  Tumor size: 2.1 cm in horizontal extent  Depth of invasion: 10 / 22 mm cervical wall  No extension to bilateral parametria  Lymphovascular invasion: present  Perineural invasion: not identified  Vaginal resection margin: negative  Lymph node metastasis: 0 out of 18  PDX tumor sample acquired.  Adjuvant radiotherapy (*During post-diagnosis 1 and 3 months*)  Whole pelvis 50.4 Gy / 28 fractions  Last follow-up 93 months after initial diagnosis with no evidence of disease recurrence |
| II | Primary  squamous cell  carcinoma | 59 | IB3 | Radical abdominal hysterectomy, bilateral sapingo-oophorectomy and pelvic lymphadenectomy  Invasive squamous cell carcinoma, non-keratinizing type  Tumor size: 4.4 x 1.8 cm  Depth of invasion: 14 / 16 mm cervical wall  Involvement of uterine corpus (endometrium/myometrium): absent  No extension to bilateral parametria  Lymphovascular invasion: negative  Vaginal resection margin: negative (safety margin: 1 mm)  Lymph node metastasis: 0 out of 19  PDX tumor sample acquired.  Adjuvant radiotherapy (*During post-diagnosis 1 and 3 months*)  Whole pelvis 50.4 Gy / 28 fractions  Ovoid boost 3.0 Gy / 6 fractions (due to close resection margin)  Last follow-up 81 months after initial diagnosis with no evidence of disease recurrence |
| III | Primary  adenocarcinoma | 45 | IIB | Laparoscopic radical hysterectomy, bilateral salpingo-oophorectomy and pelvic lymphadenectomy  Adenocarcinoma, endocervical type  Tumor size: 4.5 x 2.5 cm  Depth of invasion: 20 / 22 mm cervical wall  Involvement of uterine corpus (endometrium/myometrium): absent  Parametrial invasion: present (right)  Lymphovascular invasion: negative  Vaginal resection margin: negative  Lymph node metastasis: 0 out of 15  PDX tumor sample acquired.  Adjuvant concurrent chemoradiation therapy (*During post-diagnosis 1 and 2 months*)  Weekly-cisplatin three times (40 mg/m^2^)  Whole pelvis 40.0 Gy / 16 fractions |
| IV | Recurrent  squamous cell  carcinoma | 52 | IIIB | Last follow-up 30 months after initial diagnosis with no evidence of disease recurrence  Definitive concurrent chemoradiation therapy (*During post-diagnosis 1 and 2 months*)  Weekly-cisplatin four times (40 mg/m^2^)  Whole pelvis 30.6 Gy / 17 fractions  Treatment discontinued due to poor general conditions  Disease progression seen on imaging follow-up (*Post-diagnosis 13 months*)  From 3.7 cm to 5.4 cm  Total abdominal hysterectomy, bilateral salpingo-oophorectomy and adhesiolysis (*Post-diagnosis 15 months*)  Invasive squamous cell carcinoma, non-keratinizing type  Tumor size: 5.0 x 1.6 cm  Depth of invasion: 20 / 22 mm of myometrial wall  Involvement of uterine corpus (myometrium): present  Parametrial invasion: unable to evaluate  Vaginal resection margins by invasive carcinoma: present  Lymphovascular invasion: positive  Metastatic carcinoma: left ovary and peritoneum  PDX tumor sample acquired.  Paclitaxel-cisplatin-bevacizumab #6 (*During post-diagnosis 16 and 19 months*)  Recurrent tumor at the vaginal stump seen on imaging follow-up (*Post-diagnosis 35 months*)  2.3 cm at the right side vaginal stump invading right distal ureter  Salvage radiotherapy (intensity modulated proton beam therapy) (*During post-diagnosis 36 and 38 months*)  Stump mass 60.0 Gy / 30 fractions  Last follow-up 50 months after initial diagnosis with no evidence of disease recurrence |
| V | Recurrent  adenocarcinoma | 33 | IIIC1 | Robot laparoscopic radical hysterectomy, right salpingectomy, left salpingo-oophorectomy,  bilateral pelvic lymphadenectomy, bilateral paraaortic lymphadenectomy, right ovary transposition  Adenocarcinoma, endocervical type with focal squamous differentiation  Tumor size: 3.5 x 3.5 cm  Depth of invasion: 4 / 10 mm cervical wall  Involvement of uterine corpus (endometrium/myometrium): present  Involvement of upper vagina: present  Vaginal resection margins: negative  Parametrial invasion: absent  Lymphovascular invasion: positive  Lymph node metastasis: 5 out of 14 pelvic lymph nodes, 0 out of 5 paraaortic lymph nodes  Adjuvant concurrent chemoradiation therapy (*During post-diagnosis 1 and 2 months*)  Weekly-cisplatin six times (40 mg/m^2^)  Whole pelvis 50.4 Gy / 28 fractions  Newly developed tumor at the left distal ureter seen on APCT (*Post-diagnosis 4 months*)  Left partial cystectomy and ureteroneocystostomy (*Post-diagnosis 5 months*)  Paclitaxel-cisplatin-bevacizumab #6 (*During post-diagnosis 6 and 10 months*)  Increase in size of tumor at the right distal ureter seen on imaging follow-up (*Post-diagnosis 17 months*)  Fluorouracil-cisplatin #2 (*During post-diagnosis 19 and 20 months*)  Increase in size of tumor at the right distal ureter seen on imaging follow-up (*Post-diagnosis 21 months*)  Topotecan-cisplatin #1 (*Post-diagnosis 21 months*)  Pelvic mass excision, total cystectomy, ileal conduit, lower anterior resection, ileostomy (*Post-diagnosis 22 months*)  PDX tumor sample acquired.  Salvage radiotherapy (intensity modulated proton bean therapy (*During post-diagnosis 25 and 26 months*)  Right pelvic residual tumor 60.0 Gy / 25 fractions  Treatment discontinued due to poor general conditions  Patient died of the disease 32 months after her initial diagnosis |

^a^PDX: Patient-derived xenograft.

^b^FIGO (International Federation of Gynecology and Obstetrics) stages (2018).
