# Supplementary material for: Anti-Cancer Activity of As4O6 and its Efficacy in a Series of Patient-Derived Xenografts for Human Cervical Cancer
Source: Pharmaceutics. 2020 Oct 19;12(10):987. doi: 10.3390/pharmaceutics12100987 (PMC7590205; doi:10.3390/pharmaceutics12100987)

Supplementary Figure 1. T2 sagittal magnetic resonance imaging (MRI) of the cancer lesion that was used to generate the patient-derived xenograft (PDX) I. The main lesion is located on the anterior lip of the cervix, which is measured at 2.2 cm.

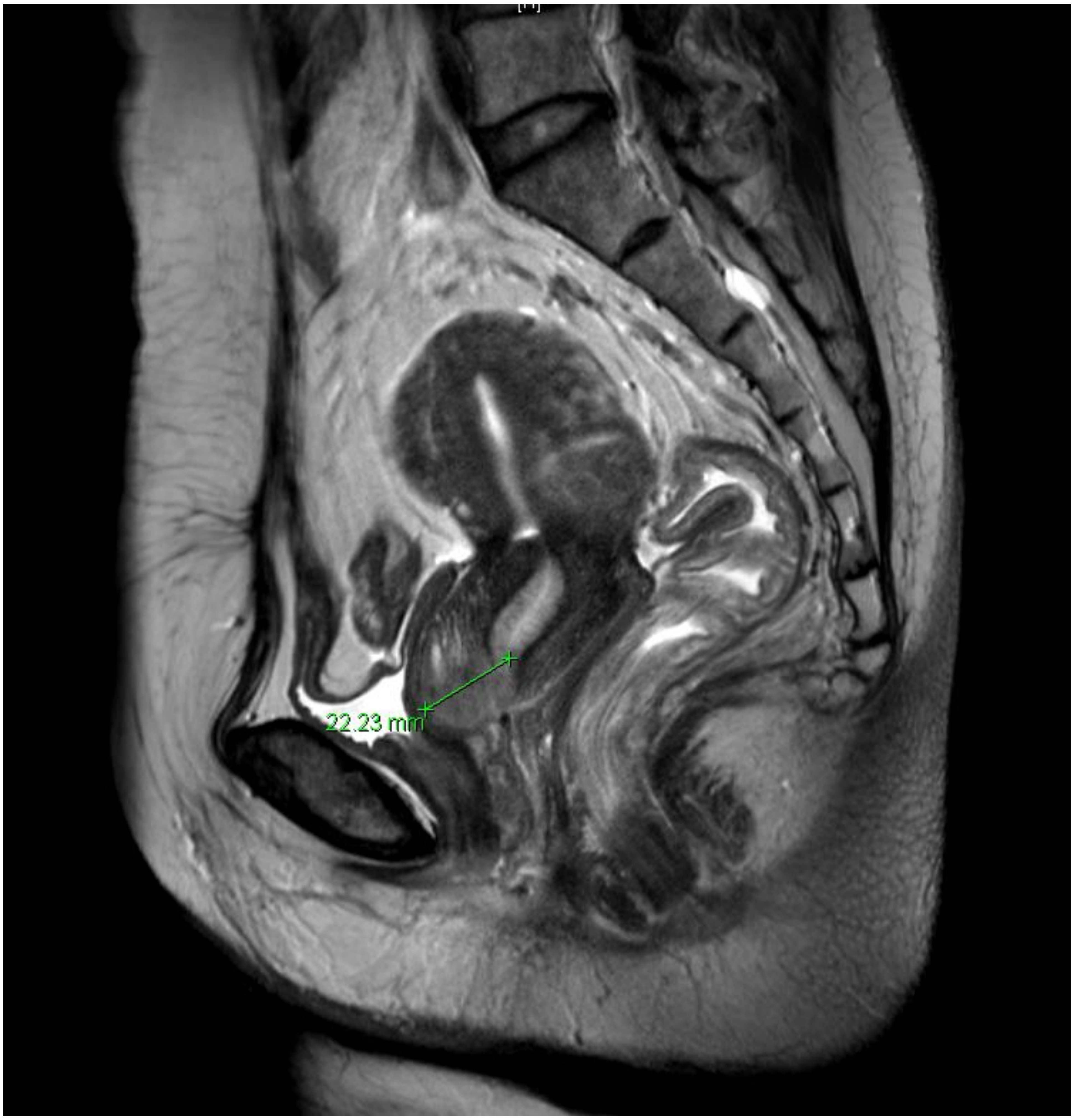

Supplementary Figure 2. T2 sagittal MRI of the cancer lesion that was used to generate the PDX II. The mass is located on the posterior lip of the cervix and measured at 4.3 cm at its longest dimension.

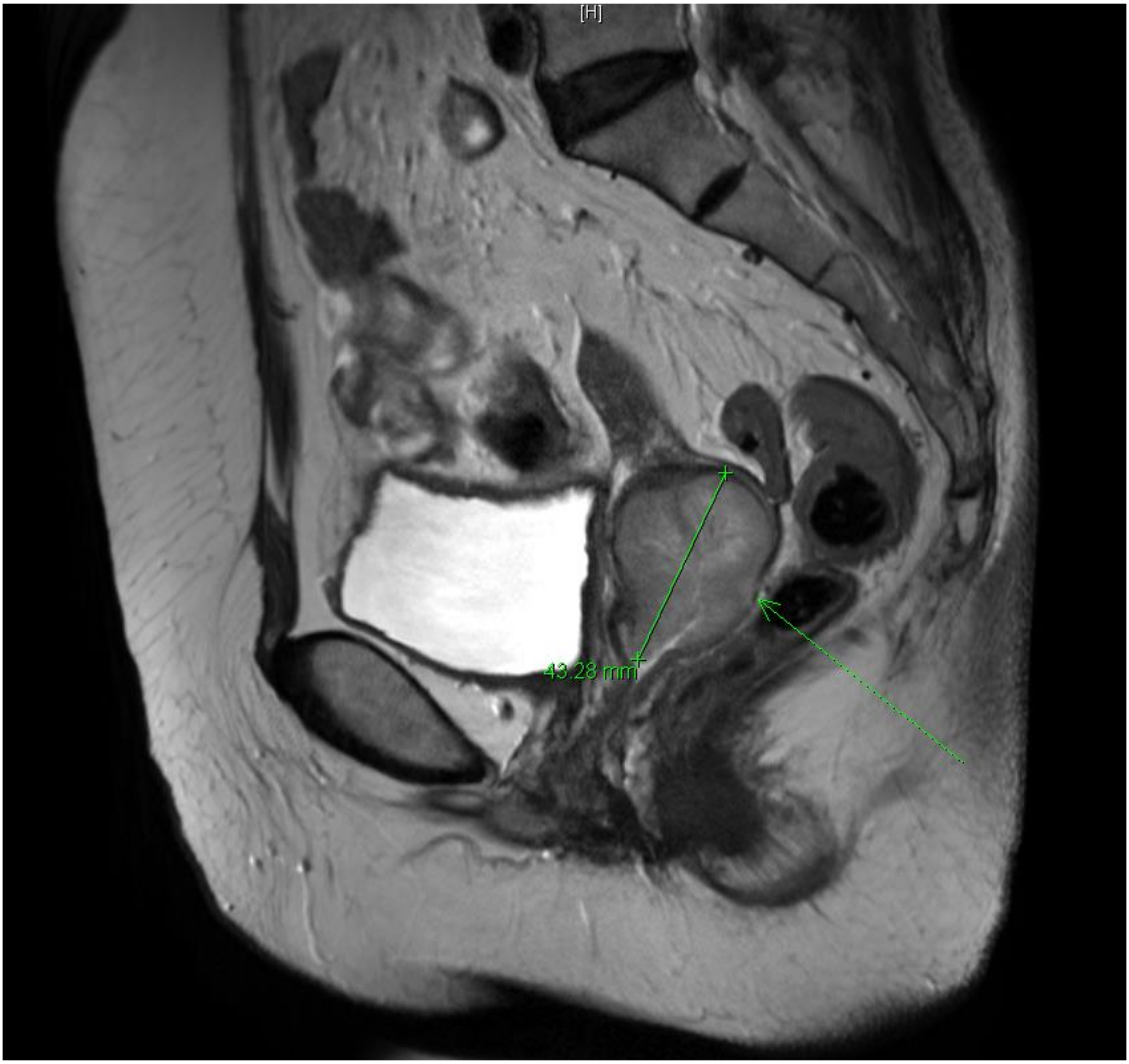

Supplementary Figure 3. T2 sagittal MRI of the cancer lesion that was used to generate the PDX III. A 4.9 cm mass is located on the anterior lip of the cervix.

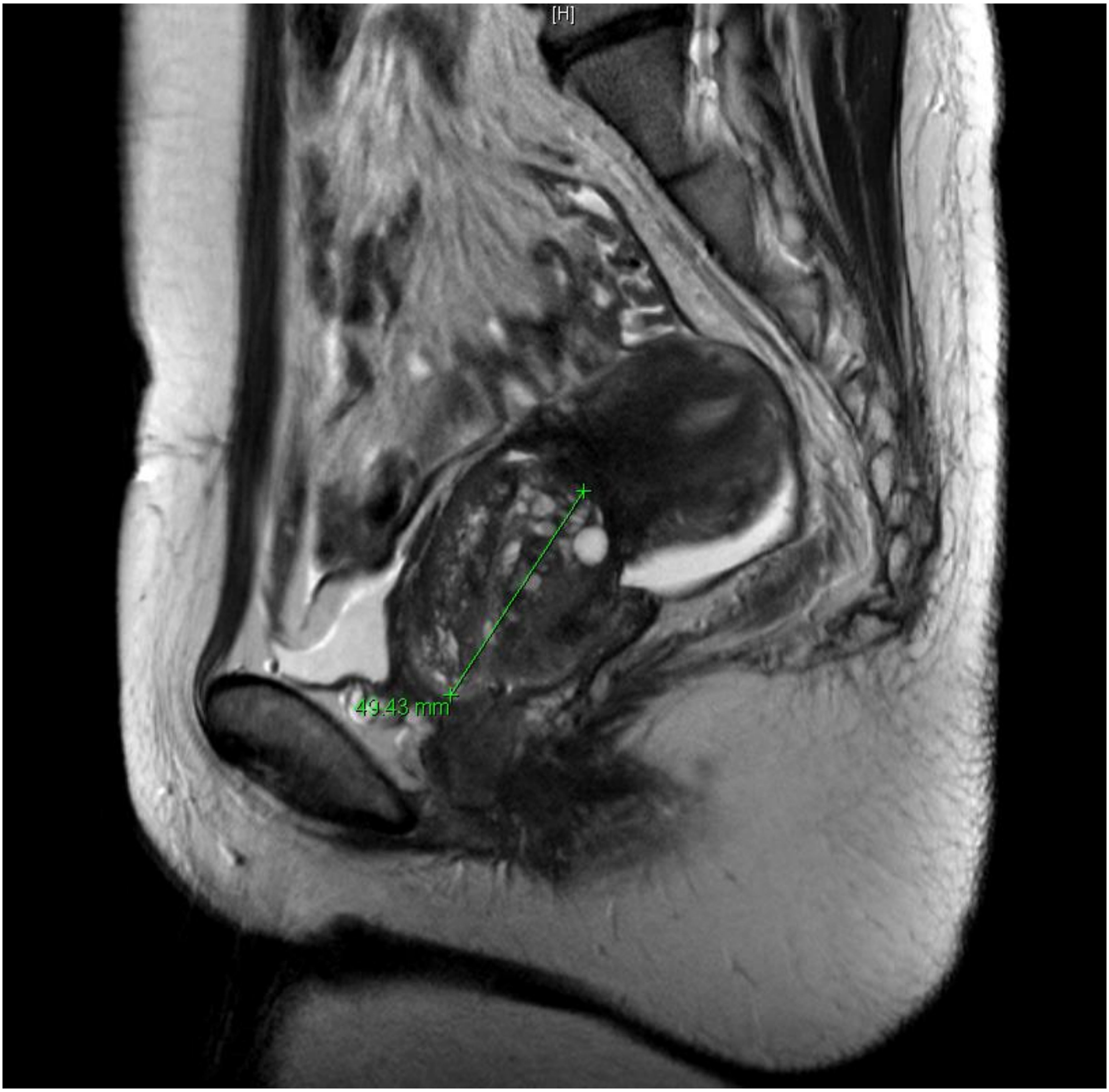

Supplementary Figure 4. T2 transverse MRI of the cancer lesion that was used to generate the PDX IV (top). A 5.4 cm mass is seen on the posterior lip of the cervix. The same lesion exhibits increased FDG (^{18}F-fluorodeoxyglucose) uptake (bottom).

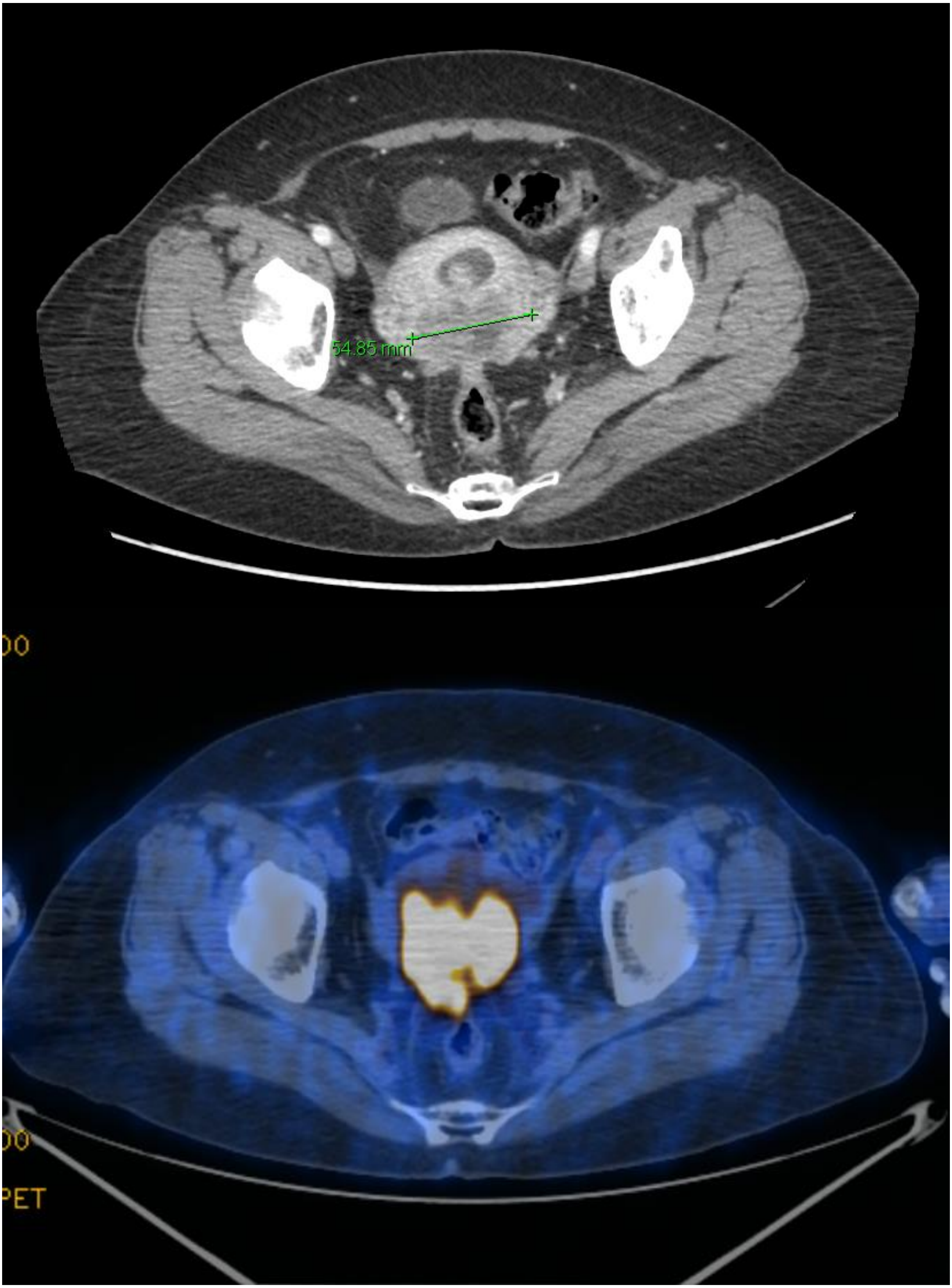

Supplementary Figure 5. T2 transverse MRI of the cancer lesion that was used to generate the PDX V (top). A 4.1 cm mass is seen on the right side of the vaginal stump. The same lesion exhibits increased FDG uptake (bottom).

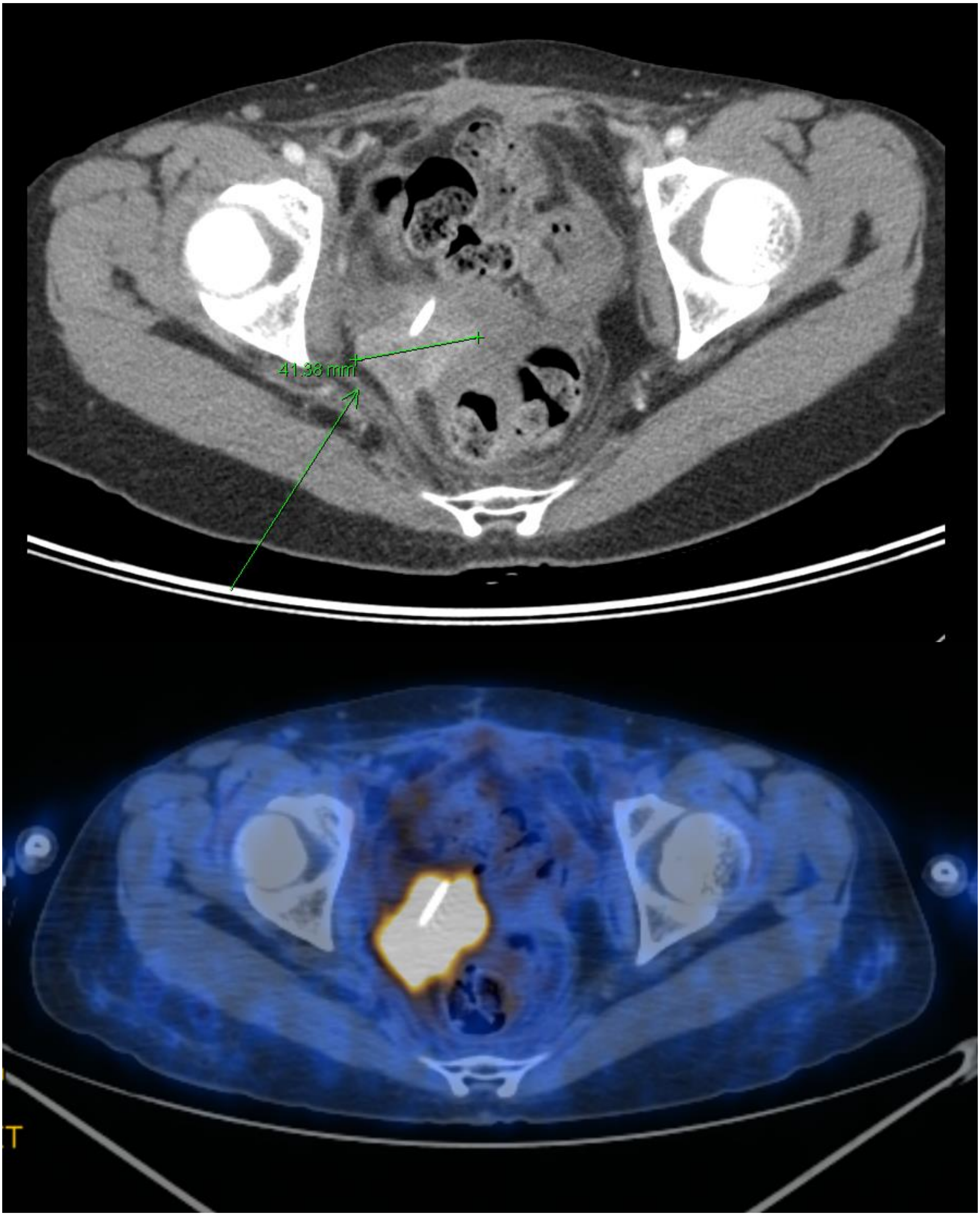

Supplement: Supplementary file 1 [file pharmaceutics-12-00987-s001.zip › pharmacuetics 958758 supplementary proof/supplementary figure.pdf]
